# Supplementary material for: Dosage differences in 12-OXOPHYTODIENOATE REDUCTASE genes modulate wheat root growth
Source: Nat Commun. 2023 Feb 1;14:539. doi: 10.1038/s41467-023-36248-y (PMC9892559; doi:10.1038/s41467-023-36248-y)
Supplement: Supplementary file 1 — Supplementary Information [file 41467_2023_36248_MOESM1_ESM.pdf]

Dosage differences in *12-OXOPHYTODIENOATE REDUCTASE* genes modulate wheat root growth

Gabay *et al.*

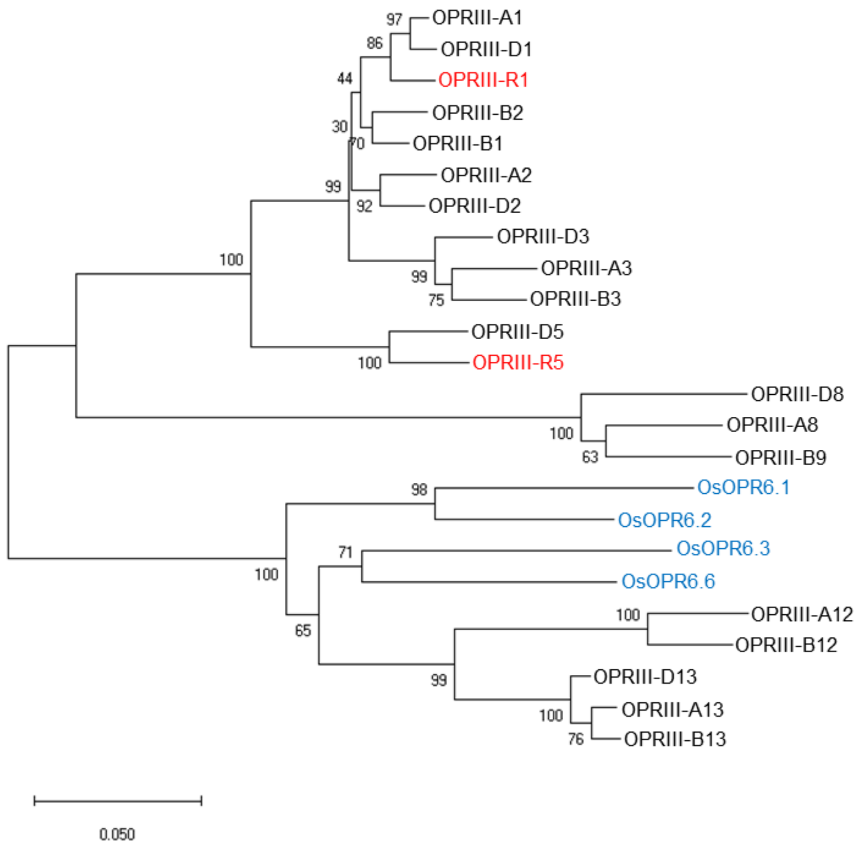

Notes:
OPRIII-B2 is more closely related to OPRIII1 than to OPRIII2 proteins, and may need to be renamed. OPRIII genes in 1BS are organized in a different order than in 1AS or 1DS suggesting some rearrangements.

OPRIII-B9 is closely related to the OPRIII8 proteins and may need to be renamed.

OPRIII12 and OPRIII13 located in wheat homeologous group 7 clustered with rice OsOPR6 genes in a colinear region on rice chromosome 6. The other wheat and rye OPRIII genes located in the duplicated region in the short arm of homoeologous group 1 form a separate cluster with no orthologs in rice. A similar clustering has been described in [1]

Tree in Newick machine-readable format: ((((((OPRIII-A1,OPRIII-D1),OPRIII-R1),(OPRIII-B2,OPRIII-B1)),(OPRIII-A2,OPRIII-D2)),(OPRIII-D3,(OPRIII-A3,OPRIII-B3))),(OPRIII-D5,OPRIII-R5)),(OPR-D8,(OPR-A8,OPR-B9))),((OsOPR6.1,OsOPR6.2),((OsOPR6.3,OsOPR6.6),((OPRIII-A12,OPRIII-B12),(OPRIII-D13,(OPRIII-A13,OPRIII-B13))))));

Supplementary Figure 1. Phylogenetic relationship among OPRIII proteins in rye, wheat and rice. We inferred the evolutionary history of OPRIII proteins from rye, wheat and rice using the Neighbor-Joining method. We used the MUSCLE protein alignment presented in the next page to calculate the optimal tree with branch lengths proportional to the distances used to infer the phylogenetic tree. The evolutionary distances were computed using the Poisson correction method, and are in the units of the number of amino acid substitutions per site. We show the percentage of replicate trees in which the associated taxa clustered together in the bootstrap test (1000 replicates) next to the branches. We removed all ambiguous positions for each sequence pair (pairwise deletion option) and the final dataset included 401 positions. We conducted all the analyses using MEGA X [2]. OPR nomenclature is based on Mou *et al.* [1]. These authors also provide a more complete phylogenetic analysis of all wheat OPR proteins. We used this analysis to assign names to the rye proteins based on their relationship with the named wheat genes (in red). Source data are provided as a Source Data file.

```
             ....:...10....:...20....:...30....:...40....:...50....:...60....:...70....:...80....:
OPRIII-A3    ------------MEPIPLLTPYK-----MGQFDLAHRVVLAPLTRRRSYANVPQPHAAVYYSQRATAGGLLIAEATVVSDTGRGY
OPRIII-B3    ------------MEPIPLLTPYK-----MGQFDLAHRVVLAPLTRRRSYANVPQPHAAVYYSQRATAGGLLIAEATVISDTGRGY
OPRIII-D3    ------------MEPIPLLTPYK-----MGQFDLAHRVVLAPLTRRRSYANMPQPHAAVYYSQRATAGGLLIAEATVVSNTGRGY
OPRIII-A2    ------------MEPIPLLTPYK-----MGQFDLAHRVVLAPLTRQRSYGNVPQPHAAVYYSQRATAGGLLIAEATGVSDTAQGY
OPRIII-D2    ------------MEPIPLLTPYK-----MGQFDLAHRVVLAPLTRQRSYGNVPQTHAAVYYSQRATTGGLLIAEATGVSDTAQGY
OPRIII-B2    ------------MEPIPLLTPYK-----MGQFDLAHRVVMAPLTRQRSYGNVPQPHAAVYYGQRATAAGLLIAEATGVSDTAQGY
OPRIII-B1    ------------MEPIPLLTPYK-----MGHFDLAHRVVLAPLTRQRSYGNVPQLHAAVYYSQRATAGGLLIAEATGISDTAQGY
OPRIII-A1    ------------MEPIPLLTPYK-----MGQFDLAHRVVLAPLTRQRSYGNVPQPHAAVYYSQRATAGGLLIAEATGVSDTAQGY
OPRIII-D1    ------------MEPIPLLTPYK-----MGQFDLAHRVVLAPLTRQRSYGNVPQSHAAVYYSQRATAGGLLIAEATGVSDTAQGY
OPRIII-R1    ------------MEPIPLLTPYK-----MGQFDLAHRVVMAPLTRQRSYGNVPQPHAAVYYSQRATAGGLLIAEATGVSDTAQGY
OPRIII-D5    ------------MEPTPLLTPYK-----MGQLDLAHRIVLAPLTRQRSYGNVPQPHAAVYYSQRATVGGLLITEATGVSDTAQGY
OPRIII-R5    ------------MEPIPLLTPYK-----MGQLDLAHRIVLAPLTRQRSYGNVPQPHAAVYYSQRASAGGLLITEATGVSDTAQGY
OPRIII-A8    ---MAGEEGETGAAAIPLLAPYRAG---SGELELAHRVVLAPLTRQRSPGNLPQPHAAVYYAQRATAGGMLITEATGVSAAAQGH
OPRIII-D8    MAGEDGETVTVAAAAIPLLTPYRTG---GGELELAHRVVLAPLTRQRSPGNLPQPHAAVYYAQRATAGGMLITEATGVSAAAQGH
OPRIII-B9    ------------------------------------------MTRQRSPGNLPQPHAAVYYAQRATAGGMLITEATGVSAAAQGH
OPRIII-A12   ---------MATKEPIPLLTPHK-----MGQFELSHRVVLAPLTRCRSYANVPQPHAALHYSQRATKGGLLIAEATGVSATAQGF
OPRIII-B12   ---------MATKEPIPLLTPHK-----MGQFELSHRVVLAPLTRCRSYGNVPQPHAALHYSQRATKGGLLIAEATGVSATAQGF
OPRIII-A13   ---------MVAKEAIPLLTPHR-----MGRFELSHRVVLAPLTRCRSYANVPQPHAAAYYSQRATKGGLLIAEATGVSATAQGY
OPRIII-B13   ---------MVAKEAIPLLTPHR-----MGRFELSHRVVLAPLTRCRSYANVPQPHAALYYSQRATEGGLLIAEATGVSATAQGY
OPRIII-D13   ---------MVAKEAIPLLTPHK-----MGQFELSHRVVLAPLTRCRSYANVPQPHAAAYYSQRATRGGLLIAEATGVSATAQGY
OsOPR6.1     MVQHHQAAANDDHQAIPLLTPYKQAGRPGSKLDLSHRVLLAPMTRCRSYGNVPQPHAALYYTQRATRGGLLITEATGVSATAQGY
OsOPR6.2     ---------MVNQAAMPLLTPYKQA---GGKIDLSHRVLLSPMTRCRSYGNVPQPHAALYYTQRATSGGLLITEATGVSDTAQGY
OsOPR6.3     -MAREAEKDAAAAAEIPLLTPYK-----MGRFELSHRVVLAPLTRNRSYGNVPRPHAVLYYTQRATSGGLLVTEATGVSDTAQGY
OsOPR6.6     --MVHAPAKVAAAAAIPLLTPYK-----MGQLELSHRVVLAPLTRCRSYGNVPQPHAAVYYSQRATRGGLLIAEATDISPTAQGY

             ...90....:..100....:..110....:..120....:..130....:..140....:..150....:..160....:..170
OPRIII-A3    TDTPGIWTAEHVEAWKPIVTAVHAKGALFFCQLWHVGRVSTFELQPGGAA-----PLSSTEKGVGPQLSFDGRLEEFSPPRRLTV
OPRIII-B3    TDTPGIWTAEHVEAWKPIIAAVHAKGALFFCQLWHVGRVSTFELQPGGAA-----PLSSTEKGVGPQMTFDGRLEEFSPPRRLTV
OPRIII-D3    TDTPGIWTAEHVEAWKPIVAAVHAKGALFFCQIWHVGRVSTFELQPGGAA-----PLSSTEKGVGPQMSFDGRLEEFSPPRRLTV
OPRIII-A2    TDTPGIWTTEHIEAWKPIVAAVHAKGALFFCQIWHVGRVSTFELQPGGAA-----PLSSTEKGVGPQISFDGHREEFSPPRRLTV
OPRIII-D2    TDTPGIWTAEHVEAWKPIVAAVHAKGALFFCQIWHVGRVSTFELQPGGAA-----PLSSTEKGVGPQMSFDGHREEFSPPRRLTV
OPRIII-B2    TDTPGIWTAEHVEAWKPIVAAVHAKGALFFCQIWHVGRVSTFELQPGGAA-----PLSSTEKGVGPQMSFDGRLEEFSPPRRLTV
OPRIII-B1    TDTPGIWTAEHVEAWKPIVAAVHAKGALFFCQIWHVGRVSTFELQPGGAA-----PLSSTEKGVGPQMSFDGRLEEFSPPRRLTV
OPRIII-A1    NDTPGIWTAEHVEAWKPIVAAVHQKGALFFCQIWHCGRVSTFELQPGGTA-----PLSSTEKGVGPQMSFDGRLEEFAPPRRLTV
OPRIII-D1    NDTPGIWTAEHVEAWKPIVAAVHQKGALFFCQIWHCGRVSTFELQPGGTA-----PLSSTEKGVGPQMSFDGRLEEFAPPRRLTV
OPRIII-R1    NDTPGIWTAEHVEAWKPIVAAVHAKGALFFCQIWHCGRVSTFELQPGGTA-----PLSSTEKGVGPQMSFDGRLEEFAPPRRLTV
OPRIII-D5    RDTPGVWTAEHVEAWKPIVDAVHAKGALIFCQIWHVGRVSTYEYQPGGAA-----PLSCTDKGVGPQMSYDGRLEEFAPPRRLKV
OPRIII-R5    RDTPGVWTAEHVEAWKPIIDAVHAKGAIIFCQIWHVGRVSTYEYQPGGAA-----PLSCTDKGVGPQMSYDGRLEEFAPPRRLTV
OPRIII-A8    RPTPGVWTDEQVDAWRPVVDAVHAKGAVIFCQLWHVGRVVGK-LRPDGTPAGTPRPVSSTGRPIATPRMNDGVEEEFATPRRLDV
OPRIII-D8    RPTPGVWAGEQVEAWRPVVDAVHAKGAVVFCQLWHVGRVVGK-LRPDGARAETPQPVSSTGRPIATPRMNDGVEEEFATPRRLDV
OPRIII-B9    RPTPGVWTDEQVEAWRPIVDAVHTKGAVIFCQLWHVGRVVGA-LRPDGTRAETV-PVSSTGRPIATPRMNDGVEEEFATPRRLAV
OPRIII-A12   PDTPGIWTQQQVNAWKPIVDAVHSKGALFFCQIWHAGRVSSNEFQPDGHA-----PISSTDKQIPPDAE-SGMV--YSKPRRLHT
OPRIII-B12   PDTPGIWTQQQVDAWKPIVDAVHGKGALFFCQIWHAGRVSTNEFQPDGHA-----PISSTDKQIPPDAE-SDTV--YSKPRQLHT
OPRIII-A13   PETPGIWTQQQVDAWKPIVDAVHRKGALFFCQIWHVGRVSTNDFQPDGQA-----PISSTDKQITPDAE-SGMV--YSKPRQLQT
OPRIII-B13   PETPGIWTQQHVDAWKPIVDAVHRKGALFFCQIWHVGRVSTNDFQPDGQA-----PISSTDQQITPDAE-SGMV--YSKPRQLQT
OPRIII-D13   PETPGIWTQQQVDAWKPIVDAVHRKGALFFCQIWHVGRVSTNDFQPDGQA-----PISSTDKQITPDAE-SGMV--YSKPRQLQT
OsOPR6.1     PETPGVRTREHVEAWKPIVDAVHRKGALFICQLWHVGRVSNNGFQPDGLA-----PISSTDKAITPDGY--GMV--YSKPRRLRT
OsOPR6.2     PETPGVWTREHVEAWKPIVDAVHRKGALFICQLWHVGRVSTNDYQPNGQA-----PISSSDIQITPDGS--GIV--YSKPRRLRV
OsOPR6.3     PDTPGIWTQQQVEAWKPIVDAVHRKGALFICQLWHVGRVSTNEYQPDGQA-----PISSTDRQITPDDS--GIV--YSKPRRLRT
OsOPR6.6     PETPGIYTQQQIEAWKPIVDAVHRKGALFFLQIWHVGRVSTTDFQPNGQA-----PISSTDKQITPDDS--GMV—YSKPRRLRT

             ....:..180....:..190....:..200....:..210....:..220....:..230....:..240....:..250....:
OPRIII-A3    EEIPAIVDDFRKAARNAIDAGFDGVEIHAANGYIIEQFLKDSANDRTDEYGGSLENRCRFALEVVDAVVKEVGGHRVGIRLSPFT
OPRIII-B3    QEIPAIVDDFRKAARNAIDAGFDGVEIHAANGYIIEQFLKDSANDRTDEYGGSLENRCQFALEVVDAVVKEAGGHRVGIRLSPFA
OPRIII-D3    EEIPALVDDFRKAARNAIDAGFDGVEIHGANGYIIEQFLKDSANDRTDEYGGSLENRCRFALEVVDAVVKEVGGHRVGIRLSPFA
OPRIII-A2    EEIPAIVDDFRKAARNAIDAGFDGVEIHGANGYIIEQFLKDSANDRADEYGGTLENRCRFALEVVDAVVKEVGGRRVGIRLSPFT
OPRIII-D2    EEIPAIVDDFRKAARNAIDAGFDGVEIHGANGYIIEQFLKDSANDRTDEYGGSLENRCRFALEVVDAVVKEVGGRRVGIRLSPFT
OPRIII-B2    EEIPAIIDDFRKAARNAIDAGFDGVEIHGANGYIIEQFLKDSANDRTDEYGGSLENRCRFALEVVDAVVKEVGGHRVGIRLSPFT
OPRIII-B1    EEIPAIIDDFRKAARNAIDAGFDGVEIHGANGYIIEQFLKDSANDRTDEYGGSLENRCRFALEVVDAVVKEVGGHRVGIRLSPFT
OPRIII-A1    EEIPAIVDDFRKAARNAIDAGFDGVEIHGANGYIIEQFLKDSANDRIDEYGGNLENRCRFALEVVDAVVKEVGGHRVGIRLSPFT
OPRIII-D1    EEIPAIVDDFRKAARNAIDAGFDGVEIHGANGYIIEQFLKDSANDRIDEYGGSLENRCRFALEVVDAVVKEVGGHRVGIRLSPFT
OPRIII-R1    EEIPAIVDDFRKAARNVIDAGFDGVEIHGANGYIIEQFLKDSANDRTDEYGGSLENRCRFALEVVDAVIKEVGGHRVGIRLSPFT
OPRIII-D5    EEIPAIVDDFRKAARNAINAGFDGVEIHGANGYLIEQFLKDSANDRTDKYGGSIENRSRFALEVVDAVVKEVGGHRVGIRLSPFT
OPRIII-R5    EEIPAIVDDFRKAARNAIDAGFDGVEIHGANGYLIEQFLKDSANDRNDEYGGSIENRCRFALEVVDAVVKEIGGHRVGIRLSPFT
OPRIII-A8    AEIAGVVDDFRRAARNAVDAGFDGVEIHGAHGYLVEQFLKDSANDRDDEYGGSLENRCRFALEVVTAVAREIGGRRVGVRLSPFA
OPRIII-D8    AGIAGVVDDFRRAARNAVDAGFDGVEIHGAHGYLVEQFLKDSANDRDDEYGGSLENRCRFALEVVTAVAEEIGGRRLGVRLSPFA
OPRIII-B9    EEIAGVLDDFRRAARNAVYAGFDGVEIHGAHGYLVEQFLKDSTNDRDEEYGGSLENRCRFVLEVVTAVADEIGGRRVGVRLSPFA
OPRIII-A12   DEVPLIVDDFRRAARNVIEAGFDGVEIHGAHGFLLEQFMKDSSNDRTDRYGGSLENRCRFAVEVIDAIVHDIGADRVGIRLSPFT
OPRIII-B12   DEVPLIVDDFRRAARNVIEAGFDGVEIHGAHGFLLEQFMKDSSNDRTDMYGGSLENQCRFAVEVIDAIVHEIGADRVGIRLSPFV
OPRIII-A13   DEIPLIVDDFRRAARNAVEAGFDGVEIHGAHGYLLEQFMKDSSNDRTDEYGGSLENRCRFAVEVIDAIVHEIGADRVGIRLSPFV
OPRIII-B13   DEIPLIVDDFRRAARNAIEAGFDGVEIHGAHGYLLEQFMKDSSNDRTDEYGGSLKNRCRFAVEVIDAIVHEIGADRVGIRLSPFV
OPRIII-D13   DEIPLIVDDFRRAARNAIEAGFDGVEIHGAHGYLLEQFMKDSSNNRTDEYGGSLENRCRFAVEVIDAIVHEIGADRVGIRLSPFV
OsOPR6.1     DEIPQIVDDFRLAARNAVEAGFDGVEIHGANGYLLEQFMKDSSNDRTDEYGGSLENRCRFAVEVIDAVVGEIGAHSVGIRLSPFL
OsOPR6.2     DEIPQIVDDFRLAARNAIEAGFDGVEIHGANGYLLEQFMKDSSNDRTDEYGGSLENRCRFAVEVIDAVVGEIGAHRVGIRLSPFL
OsOPR6.3     EEIPQIIDDFRRAARNAIEAGFDGVEIHGAHGYLLEQFMKDSANDRSDEYGGSLENRCRFVVEVIDAIVAEVGAHRVGIRLSPFI
OsOPR6.6     DEIPQIIDDFRRAARNAIEAGFDGVEIHGAHGYLLEQFMKDSANDRTDEYGGSLENRCRFAVEVIDAVVAEVGAHRVGIRLSPFV
```

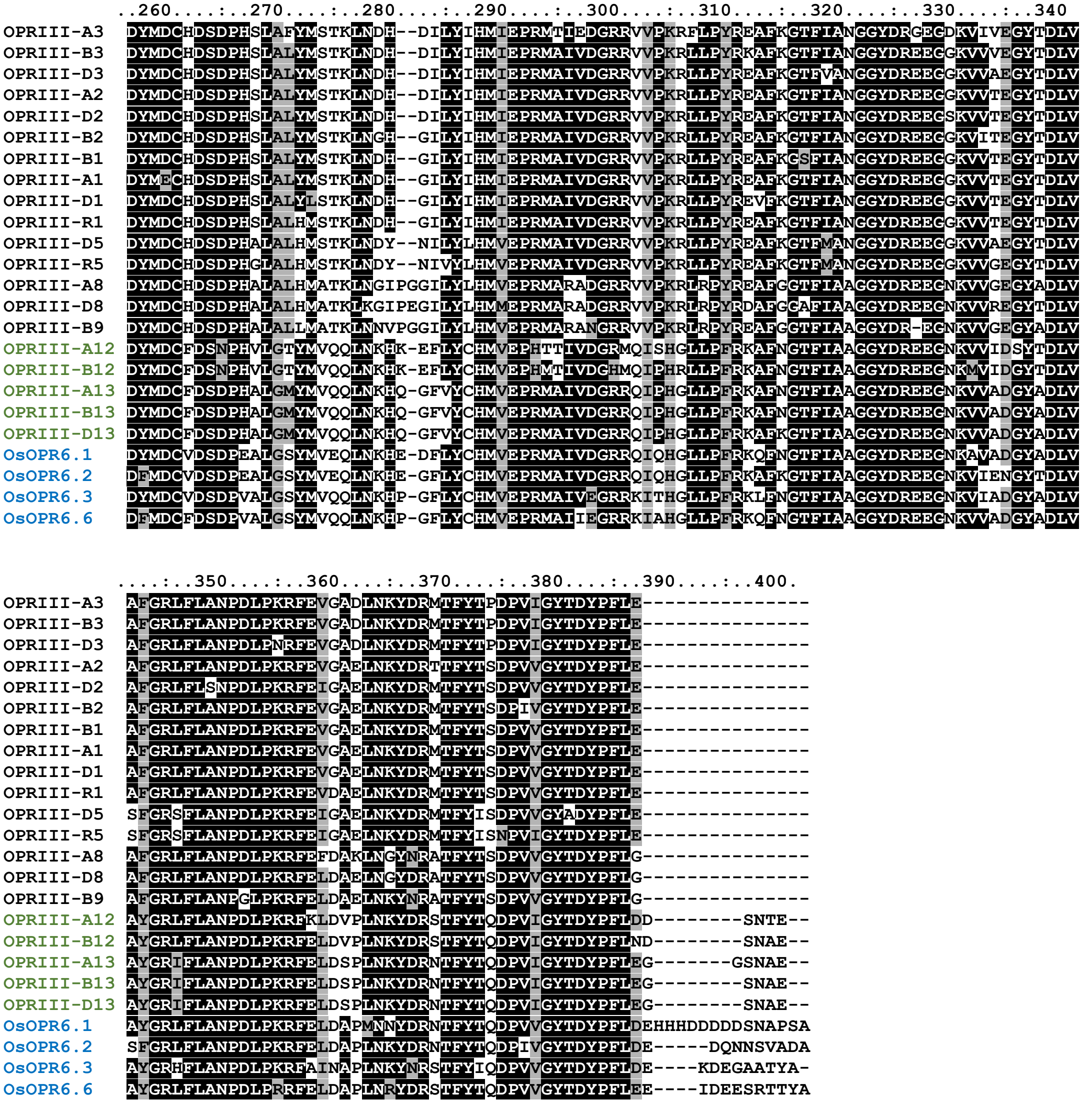

Supplementary Figure 2. The alignment of *Triticum aestivum* and *Oryza sativa* (Os) OPRIII proteins used to generate the phylogenetic tree was performed using the MUSCLE algorithm in MEGA X. The alignment was visualized using pyBoxshade (https://github.com/mdbaron42/pyBoxshade/blob/master/BS_app.py). Rice protein names are indicated in blue and the closest wheat proteins on homeologous group 7 are indicated in green. Amino acids straddling two exons (encoded by a codon split by an intron) are indicated in red. The wheat OPRIII genes located in homeologous group 7 share the same exon structure and position of straddled amino acids as the rice genes. OPRIII1, OPRIII2 and OPRIII3 proteins have three exons whereas OPRIII5, OPRIII8 and OPRIII9 have two exons. All five OPRIII genes on the short arm of homeologous group 1 share the same border between the last two exons. Source data are provided as a Source Data file

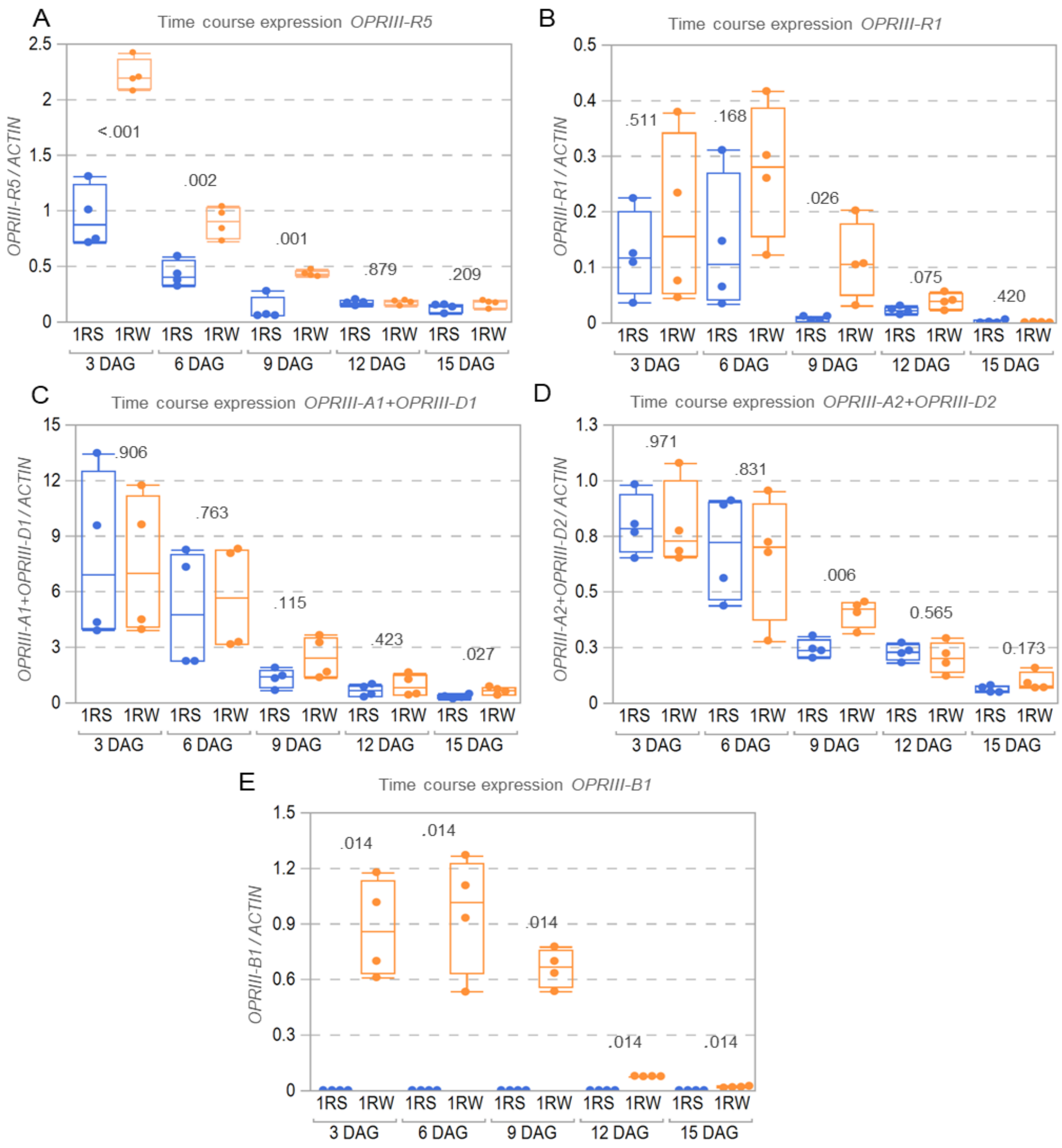

Supplementary Figure 3. Expression of *OPRIII* genes during root development. Transcript levels in the distal 1 cm of seminal roots of Hahn-1RS and Hahn-1RW collected from 3 to 15 DAG every 3 days. Rye genes (**A**) *OPRIII-R5* and (**B**) *OPRIII-R1*. Wheat genes (**C**) *OPRIII-A1* + *OPRIII-D1*, (**D**) *OPRIII-A2* + *OPRIII-D2*, and (**E**) *OPRIII-B1* (not present in 1RS). Bars indicate average transcript levels relative to *ACTIN* using the delta Ct method. Data are presented as means ± SEM. based on n = 4 at each time point for each genotype. Statistical comparisons between genotypes were perform at each time point using two-sided *t*-tests except for *OPRIII-B1*, where all 1RS values are 0 and a non-parametric two-sided Kruskal-Wallis test was used. Exact *P* values are provided on top of each comparison. The boxes in the box-plots show the range from first to third quartiles divided by the median. The whiskers span from the minimum to the maximum observation and circles indicate individual data. Source data are provided as a Source Data file.

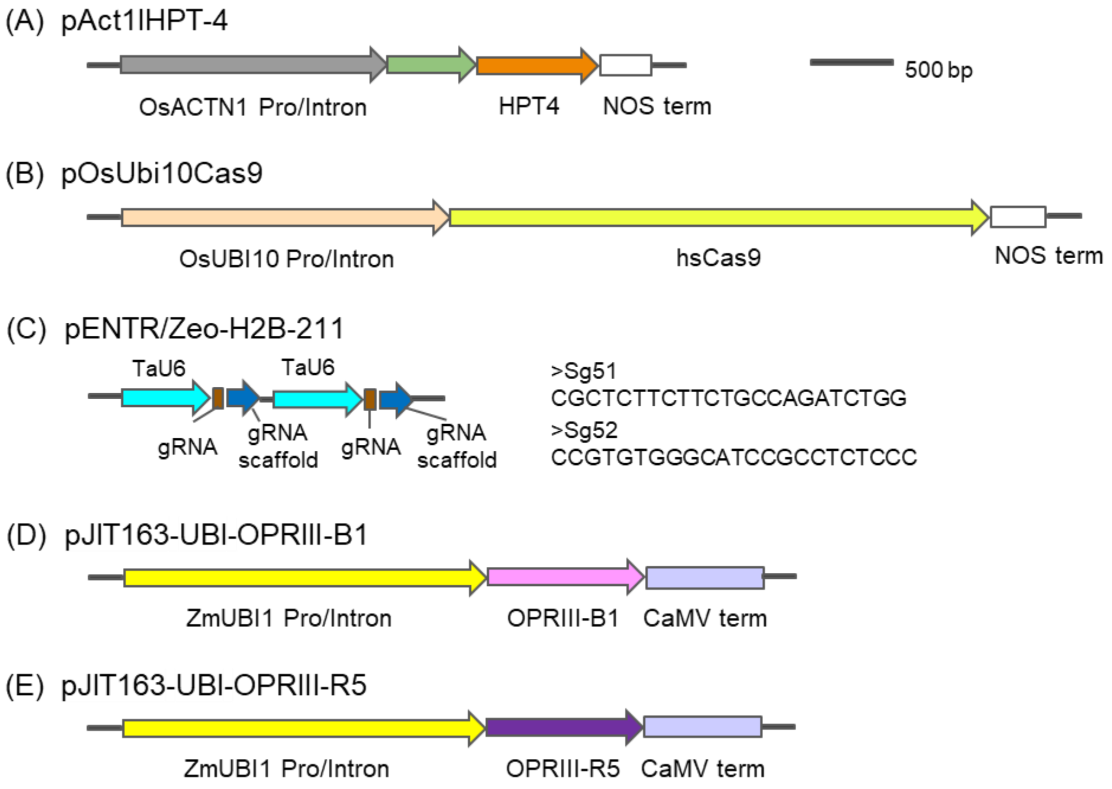

Supplementary Figure 4. Constructs used to generate transgenic Hahn 1RW plants edited for *OPRIII-B1* and Hahn-1RS plants overexpressing wheat *OPRIII-B1* and rye *OPRIII-R5* driven by the maize *UBIQUITIN1* promoter and its intron (UBI1). (**A**) pAct1IHPT-4 [3] contains hygromycin phosphotransferase (*hpt*) gene under control of the rice *ACTIN1* promoter, its intron (*act1I*) and the NOS 3' terminator. (**B**) pOsUbi10Cas9 contains the Cas9 gene from pRGEB [4] under control of the rice *UBIQUITIN10* promoter, its intron and the NOS 3' terminator. pRGEB32 was a gift from Yinong Yang (http://n2t.net/addgene:63142). (**C**) Guide RNAs Sg51 and Sg52 driven by the *T. aestivum* U6 snRNA gene (GenBank X63066.1) were cloned into vector pENTR/Zeo-H2B (GenBank GU370782). The wheat *OPRIII-B1* (**D**) and rye *OPRIII-R5* genes (**E**) were cloned in the pJIT163-UBI vector (GenBank accession LY758014.1, [5]). The *OPRIII* genes were cloned between the maize *UBIQUITIN1* promoter and the CaMV terminator (2,006 to 6,262 bp) replacing the Cas9 region. The GRF4–GIF1-CRISPR–Cas9 vector used to edit *OPRIII-R1* was described previously [6]. This experiment used the sgRNA= CTCCTCACGCCGTACAAGAT targeted to a conserved region in *OPRIII-R1*, *OPRIII-D2*, *OPRIII-A3*, *OPRIII-B3*, and *OPRIII-B2*. Source data are provided as a Source Data file.

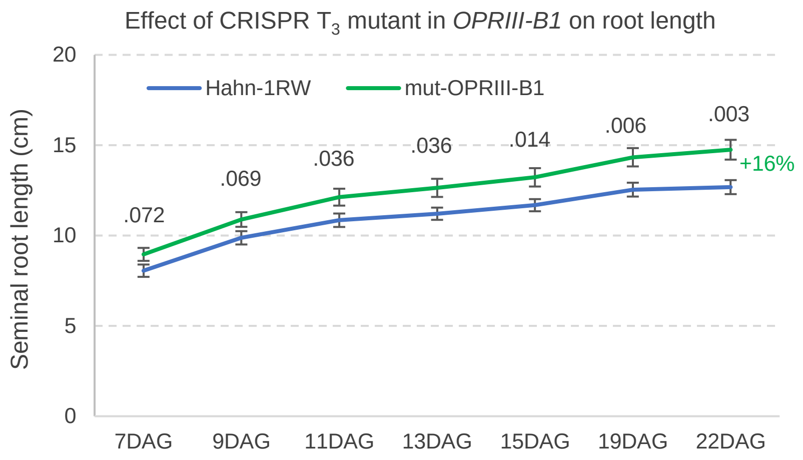

Supplementary Figure 5. Effect of a CRISPR induced 32 bp deletion in *OPRIII-B1* on root length. Time course from 7 to 22 DAG comparing 1RW T_3 sister lines with (n = 20) and without (n = 22) the 32 bp deletion in *OPRIII-B1.* A similar experiment using T_2 sister lines is presented in Fig. 2A in the main text. Data are presented as means ± SEM. *P* values were calculated using two-tailed *t*-tests at each time point. Exact *P* values are provided on top of each time point. The percent increase in root length in the mutant relative to the 1RW control is presented for the latest time point. Source data are provided as a Source Data file.

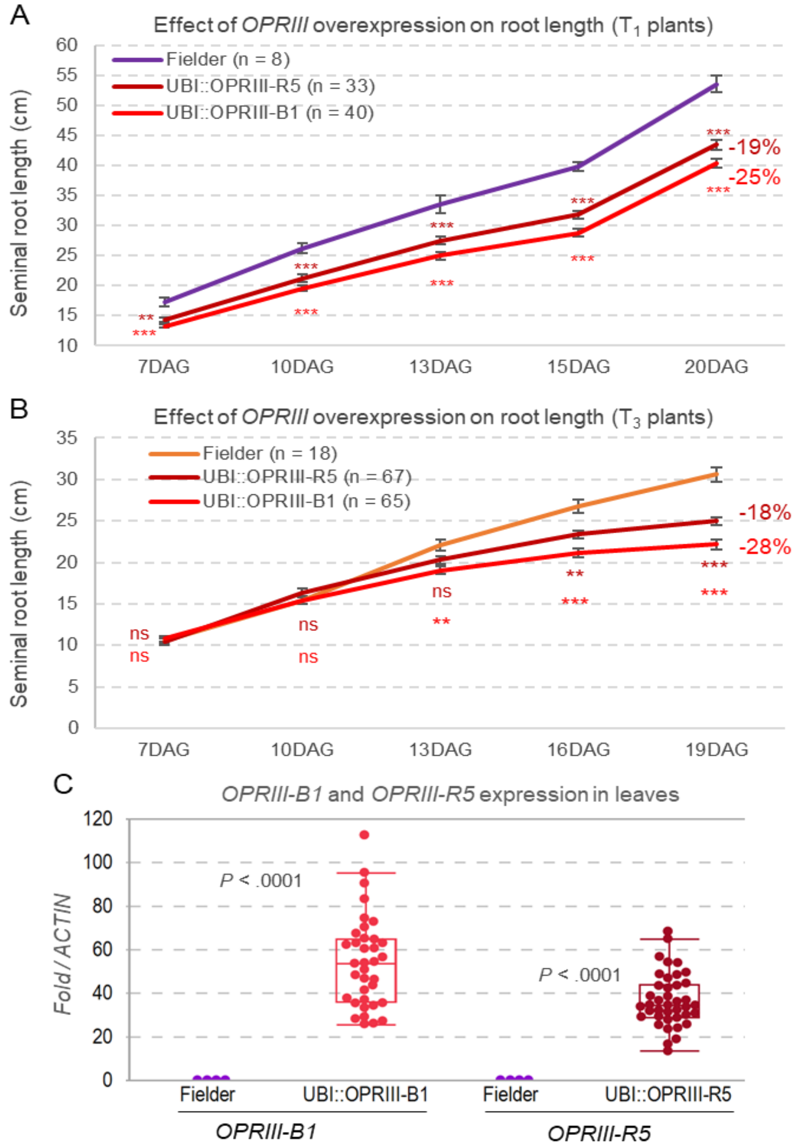

Supplementary Figure 6. Effect of overexpression of wheat *UBI::OPRIII-B1* and rye *UBI::OPRIII-R5* on root length in cultivar Fielder. (**A-B**) Time course experiment using T_1 (A) and T_3 (B) transgenic plants and sister lines without the transgenes. Data are presented as means values ± SEM. *P* values of the differences between the transgenic lines and the wildtype are based on two-sided Dunnett tests at each time point. ns = not significant, ** = $P < 0.01$ and *** = $P < 0.001$. Percent differences in root length relative to wildtype are indicated at the latest timepoint. (**C**) qRT-PCR of *OPRIII-B1* and *OPRIII-R5* relative to *ACTIN* in leaves of T_3 *UBI::OPRIII-B1* (n = 36) and *UBI::OPRIII-R5* (n = 40) transgenic plants and their respective non-transgenic controls (n = 4, endogenous genes were not detected in the Fielder control). *P* values are based on two-sided *t*-tests. The boxes in the box-plots show the range from first to third quartiles divided by the median. The whiskers span from the minimum to the maximum observation and circles indicate individual data. Source data are provided as a Source Data file.

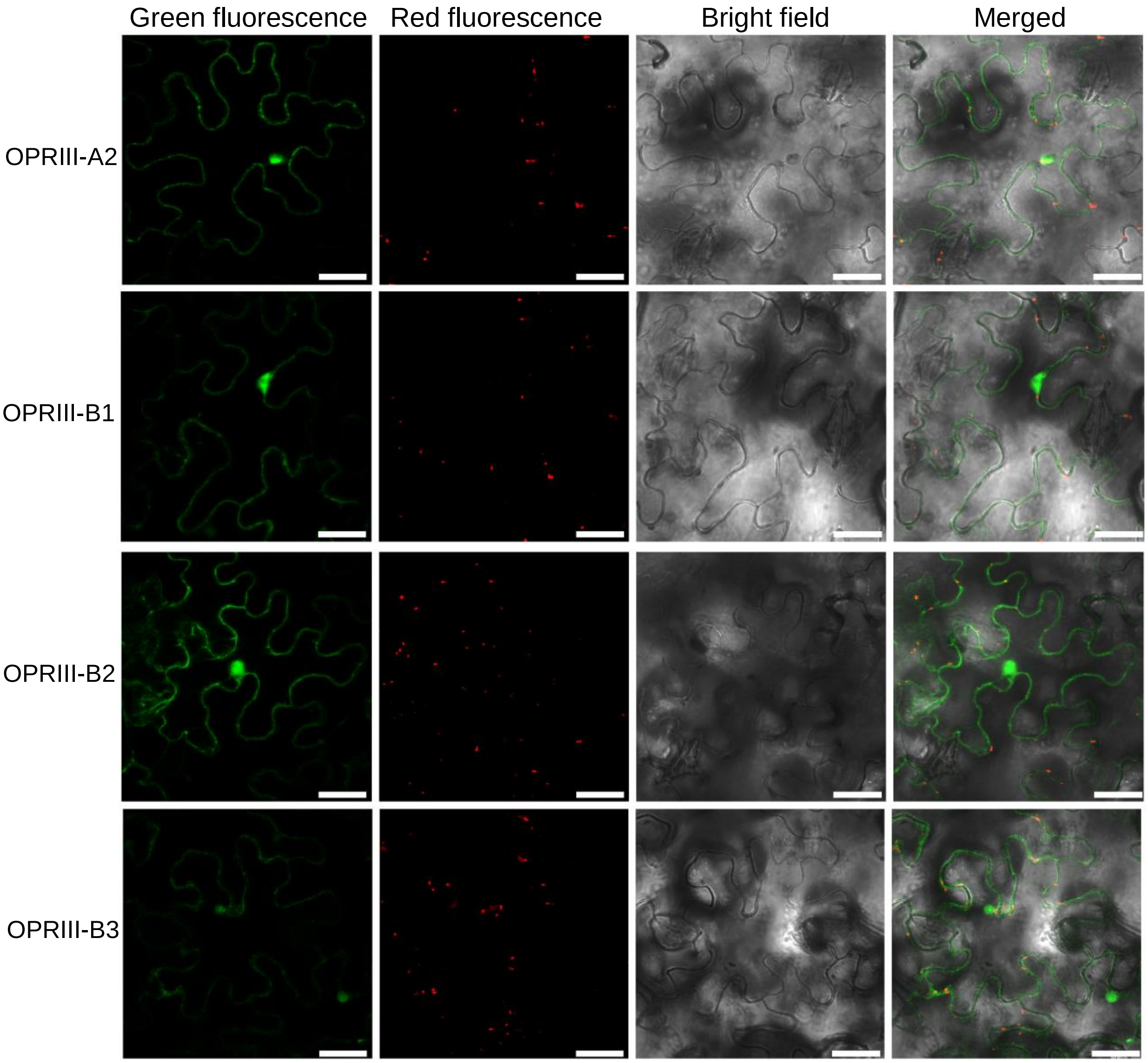

Supplementary Figure 7. Sub-cellular localization of OPRIII-A2, OPRIII-B1, OPRIII-B2, OPRIII-B3 experiment in tobacco leaves using OPRIII-GFP fusions (first column) and mCherry peroxisome marker CD3-983 (second column). The third column is the bright field and the fourth column the merged signals. Similar results were observed in multiple cells for each gene, and among the four different *OPRIII* genes. Scale bar = 10µm.

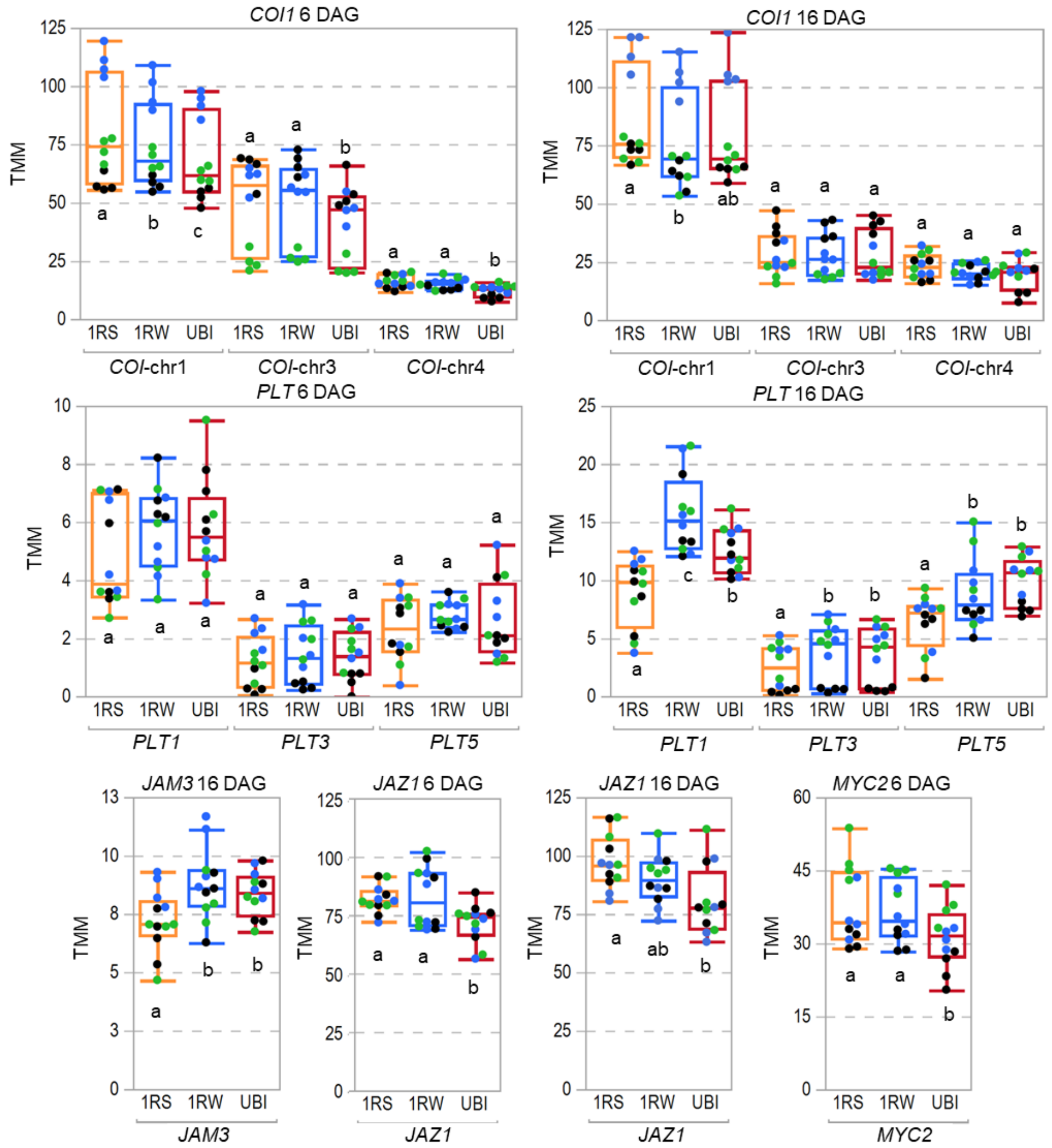

Supplementary Figure 8. Effect of higher *OPRIII* transcript levels in 1RW and UBI::OPRIII-R5 on the expression of genes from the Jasmonic Acid (JA) signaling pathways and on the JA-responsive genes *PLETHORA 1* (*PLT1*), *PLT3* and *PLT5*. From the RNA-seq data, we selected genes with consistent effects in 1RW and UBI::OPRIII-R5, and also consistent among homoeologs. We analyzed the TMM values for the three homeologs for each gene in two-sided factorial ANOVA including homoeologs and genotypes as factors and RNA-seq samples as blocks. Homeologs are presented with different dot colors: blue = A, black = B and green = D genome. Different letters indicate significant differences in Tukey tests ($P < 0.05$, n = 4) within genes. For *JAM3*, *JAZ1* and *MYC2* only the time points showing significant differences are presented. *PLT* gene numbers are based on the rice names and the similarities between the wheat and rice genes. Source data are provided as a Source Data file.

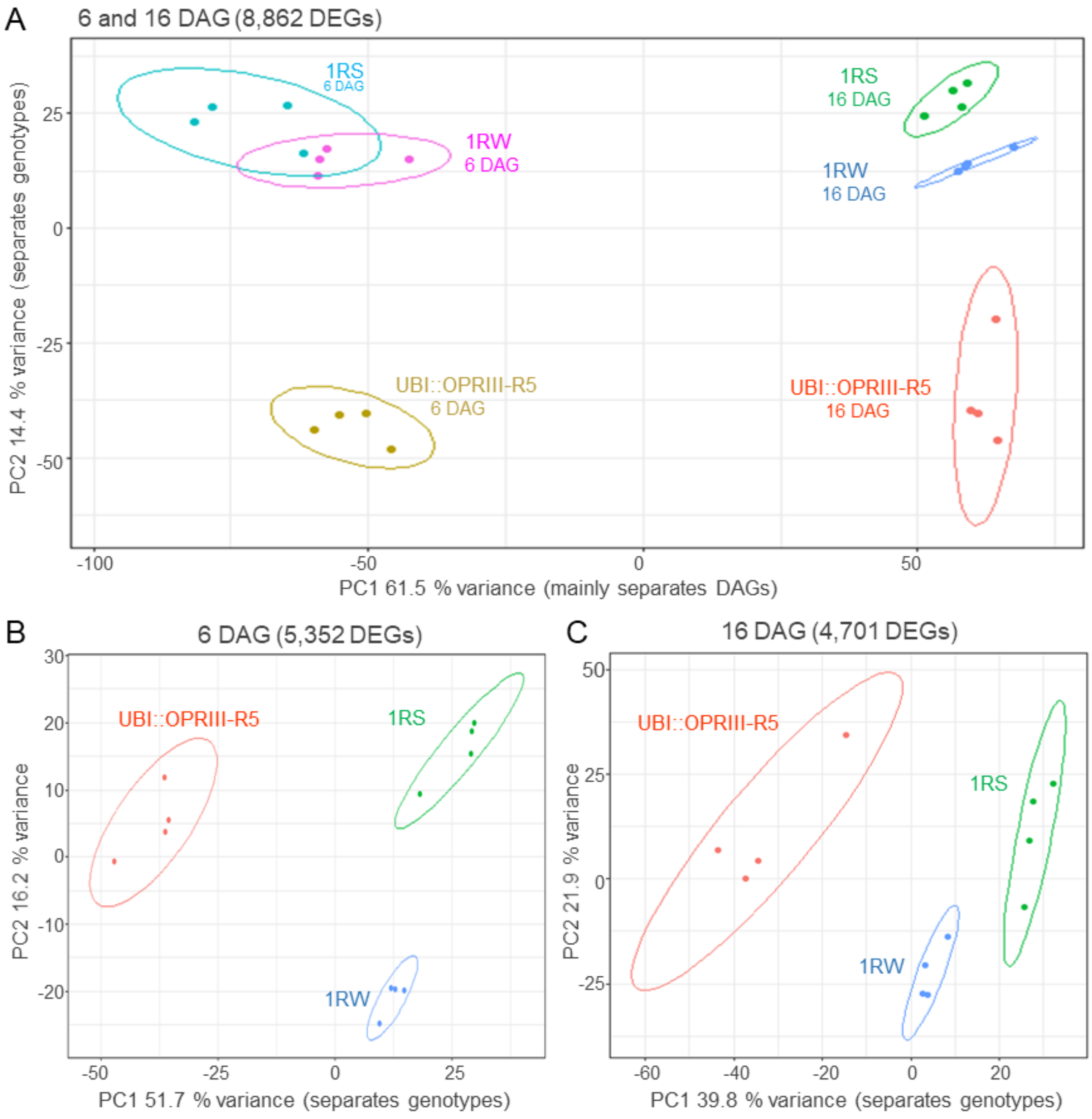

Supplementary Figure 9. Principal component analyses of the 24 RNAseq samples (3 genotypes x 2 time-points x 4 biological replications). (**A**) Analysis based on 8,862 differentially expressed genes (DEGs) between 1RW *vs*. 1RS and between UBI::OPRIII-R5 *vs*. 1RS at both 6 and 16 days after germination (DAG, Data S19). The first principal component (PC1), which explains 61.47% of the variance, mainly separates samples collected at 6 DAG from those collected at 16 DAG (developmentally regulated genes). PC2, which explains 14.41 % of the variation, mainly separates the genotypes within the same day. To visualize better the relationships among genotypes we also performed separate PCA for (**B**) 6 DAG and (**C**) 16 DAG.

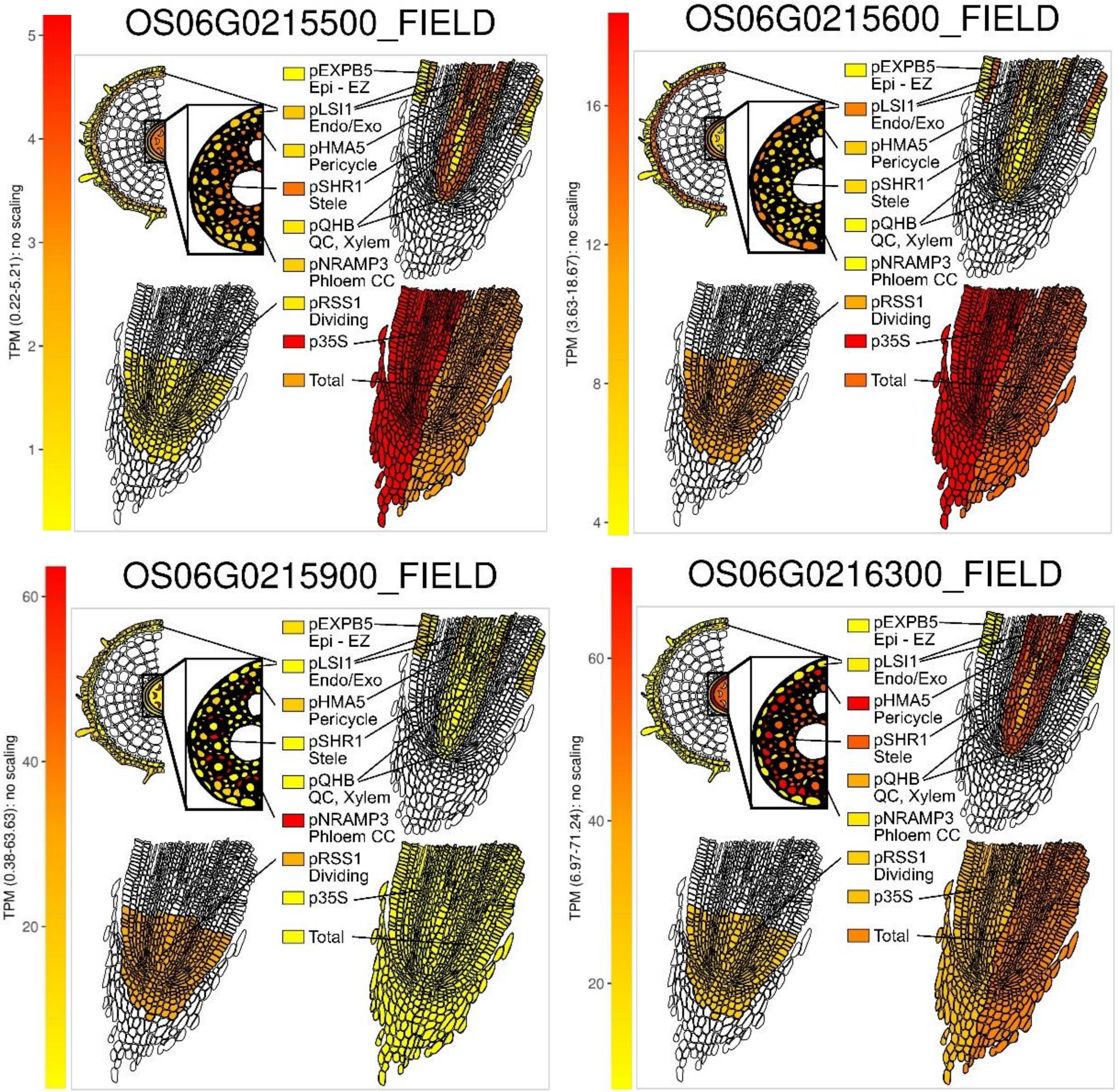

Supplementary Figure 10. Scaled transcripts per million (TPM) reads displayed in a spatial heatmap for rice *OPRIII* genes *OS06G0215500*, *OS06G0215600*, *OS06G0215900* and *OS06G0216300*. Rice translatome abundance data for specific root cell populations were visualized using the published spatialHeatMap: http://spatialheatmap.baileyserreslab.org/. Promoters used to select the specific root cell populations are indicated in the figure and described with the complete methods in the published paper [7]. Only the filed data is presented here. Additional results for plants grown in greenhouse under different drought and water flooding conditions are available in the landing page of the web site and in the published paper.

Supplementary references

1. Mou YF, Liu YY, Tian SJ, Guo QP, Wang CS, Wen SS. Genome-wide identification and characterization of the *OPR* gene family in wheat (*Triticum aestivum* L.). *Int J Mol Sci* **20**, (2019).
2. Kumar S, Stecher G, Li M, Knyaz C, Tamura K. MEGA X: Molecular evolutionary genetics analysis across computing platforms. *Mol Biol Evol* **35**, 1547-1549 (2018).
3. Cho M-J, Jiang W, Lemaux PG. Transformation of recalcitrant barley cultivars through improvement of regenerability and decreased albinism. *Plant Sci* **138**, 229-244 (1998).
4. Xie K, Minkenberg B, Yang Y. Boosting CRISPR/Cas9 multiplex editing capability with the endogenous tRNA-processing system. *Proc Natl Acad Sci USA* **112**, 3570-3575 (2015).
5. Jin S*, et al.* Rationally designed APOBEC3B cytosine base editors with improved specificity. *Mol Cell* **79**, 728-740 (2020).
6. Debernardi JM*, et al.* A GRF-GIF chimeric protein improves the regeneration efficiency of transgenic plants. *Nat Biotechnol* **38**, 1274-1279 (2020).
7. Reynoso MA*, et al.* Gene regulatory networks shape developmental plasticity of root cell types under water extremes in rice. *Dev Cell* **57**, 1177-1192 e1176 (2022).
