# Supplementary material for: Dosage differences in 12-OXOPHYTODIENOATE REDUCTASE genes modulate wheat root growth
Source: Nat Commun. 2023 Feb 1;14:539. doi: 10.1038/s41467-023-36248-y (PMC9892559; doi:10.1038/s41467-023-36248-y)
Supplement: Supplementary file 3 — Reporting Summary [file 41467_2023_36248_MOESM3_ESM.pdf]

nature portfolio

Corresponding author(s): Jorge Dubcovsky

Last updated by author(s): January 11, 2023

Reporting Summary

Nature Portfolio wishes to improve the reproducibility of the work that we publish. This form provides structure for consistency and transparency in reporting. For further information on Nature Portfolio policies, see our Editorial Policies and the Editorial Policy Checklist.

Statistics

For all statistical analyses, confirm that the following items are present in the figure legend, table legend, main text, or Methods section.

n/a	Confirmed	
☐	☒	The exact sample size (*n*) for each experimental group/condition, given as a discrete number and unit of measurement
☐	☒	A statement on whether measurements were taken from distinct samples or whether the same sample was measured repeatedly
☐	☒	The statistical test(s) used AND whether they are one- or two-sided<br>*Only common tests should be described solely by name; describe more complex techniques in the Methods section.*
☒	☐	A description of all covariates tested
☐	☒	A description of any assumptions or corrections, such as tests of normality and adjustment for multiple comparisons
☐	☒	A full description of the statistical parameters including central tendency (e.g. means) or other basic estimates (e.g. regression coefficient) AND variation (e.g. standard deviation) or associated estimates of uncertainty (e.g. confidence intervals)
☐	☒	For null hypothesis testing, the test statistic (e.g. *F*, *t*, *r*) with confidence intervals, effect sizes, degrees of freedom and *P* value noted<br>*Give P values as exact values whenever suitable.*
☒	☐	For Bayesian analysis, information on the choice of priors and Markov chain Monte Carlo settings
☒	☐	For hierarchical and complex designs, identification of the appropriate level for tests and full reporting of outcomes
☒	☐	Estimates of effect sizes (e.g. Cohen's *d*, Pearson's *r*), indicating how they were calculated

Our web collection on statistics for biologists contains articles on many of the points above.

Software and code

Policy information about availability of computer code

Data collection	Sequencing data was generated at the University of California Davis, Genomic Center using Illumina platforms and Novogen sequencers. JA, JA-ILE and OPDA concentrations were determined at the University of California Riverside, Genomic Center using LC-MS and a TQ-XS triple quadrupole mass spectrometer (Waters) coupled to an I-class UPLC system (Waters). Sub-cellular OPRIII localization pictures were taken with a Leica TCS SP8 (Leica Microsystems, Mannheim, Germany).
Data analysis	We used MUSCLE for protein alignment and inferred the OPRIII evolutionary history using the Neighbor-Joining method as implemented in MEGA version X (https://www.megasoftware.net/). Sequence alignments were visualized using pyBoxshade (https://github.com/mdbaron42/pyBoxshade/blob/master/BS_app.py). Variants in the CRISPR screen were called for each sample (demultiplexed fastq files) using CRISgo v5 (https://github.com/pinbo/CRISgo). Demultiplexed reads were mapped to potential targets using BWA v0.7.17 (https://bio-bwa.sourceforge.net/). Variants called from CRISgo were validated by visual inspection of the bam files using IGV v2.7.2 (https://software.broadinstitute.org/software/igv/). For the RNAseq analyses, we processed the raw reads using DOE-JGI BBTools (BBMap 38.79, https://sourceforge.net/projects/bbmap/) program bbduk.sh to remove Illumina adapter contamination and low-quality reads (forcetrimleft = 21 qtrim = r trimq = 10). Reads were mapped using the splicing aware STAR aligner (v2.7.0f, from the Lexogen pipeline). Mapped reads were subjected to differential expression analysis using the DESeq2 (R/Bioconductor package v3.16). A Principal Component Analysis (PCA) was carried out with pcaExplorer (R/Bioconductor package v3.16, http://bioconductor.org/packages/pcaExplorer/). For the JA, JA-Ile and OPDA analysis we used the Skyline-Daily software v22.2 (MacCoss Lab, Seattle, WA, https://skyline.ms/project/home/software/Skyline/begin.view) to detect and integrate peak areas. KEGG analyses of the transcriptome data were carried out using the Dec. 2021 release of DAVID. All statistical analyses were performed using SAS 9.4. Rice translatome abundance data for specific root cell populations were visualized using the 2022 release of the spatialHeatMap (http://spatialheatmap.baileyserreslab.org/).

For manuscripts utilizing custom algorithms or software that are central to the research but not yet described in published literature, software must be made available to editors and reviewers. We strongly encourage code deposition in a community repository (e.g. GitHub). See the Nature Portfolio guidelines for submitting code & software for further information.

Data

Policy information about availability of data

All manuscripts must include a data availability statement. This statement should provide the following information, where applicable:

- Accession codes, unique identifiers, or web links for publicly available datasets
- A description of any restrictions on data availability
- For clinical datasets or third party data, please ensure that the statement adheres to our policy

Raw data and statistics supporting all figures, supplemental figures and transcriptome analyses are provided in Data S1-26. RNA-seq data was deposited in GenBank under BioProject numbers PRJNA819072 (Hahn-1RS, https://www.ncbi.nlm.nih.gov/bioproject/PRJNA819072), PRJNA819073 (Hahn-1RW, https://www.ncbi.nlm.nih.gov/bioproject/PRJNA819073) and PRJNA819075 (Hahn-UBI::OPRIII-R5, https://www.ncbi.nlm.nih.gov/bioproject/PRJNA819075). Quant-Seq Data was deposited in GenBank under BioProject numbers PRJNA847262 (Hahn-1RW, https://www.ncbi.nlm.nih.gov/bioproject/PRJNA847262) and PRJNA847590 (mut-OPRIII-B1, https://www.ncbi.nlm.nih.gov/bioproject/PRJNA847590). Genome sequences for the wheat Chinese Spring Genome RefSeq v1.0 and the different wheat genomes sequenced in the Wheat pan-genome project can be accessed at https://wheat.pw.usda.gov/GG3/genome_browser. Read statistics, expression data for all wheat and rye 1RS genes, and a complete list of differentially expressed genes are provided in the Supplemental online data. The genetic stocks used in this study have been deposited in the National Small Grains Collection as PI 672837, PI 672838, and PI 672839.

Human research participants

Policy information about studies involving human research participants and Sex and Gender in Research.

Reporting on sex and gender	NA
Population characteristics	NA
Recruitment	NA
Ethics oversight	NA

Note that full information on the approval of the study protocol must also be provided in the manuscript.

Field-specific reporting

Please select the one below that is the best fit for your research. If you are not sure, read the appropriate sections before making your selection.

☒ Life sciences ☐ Behavioural & social sciences ☐ Ecological, evolutionary & environmental sciences

For a reference copy of the document with all sections, see nature.com/documents/nr-reporting-summary-flat.pdf

Life sciences study design

All studies must disclose on these points even when the disclosure is negative.

Sample size	Sample size indicates the number of independent plants analyzed. Technical replications were considered subsamples and averaged before the statistical analyses. We performed preliminary experiments to estimate the level of variability and to estimate the number of replications needed. We did not performed a formal power analysis to determine the number of replications, but for experiments with P values close to significant levels, we replicated the experiments and performed combined ANOVA analyses using experiments as blocks. This is indicated in the figure legends and data source file.
Data exclusions	No data exclusions
Replication	Transcriptome data was obtained in triplicate from three interconnected comparisons: 1RS vs 1RW, 1RS vs transgenic over-expressor and 1RW versus mutant. All transcriptome experiments included four biological replicates. Each replicate included a pool of roots from 8-12 individuals with the same genotype. Root staining experiments with NBT were replicated twice with identical results. Root length experiments for mutants and transgenic lines were replicated at least twice with identical results. For the sub-cellular localization, similar results were observed in multiple cells for each gene, and in all four different OPRIII genes which served as an additional level of biological replication.
Randomization	All samples were randomized. For the hydroponic experiments each tank included all the genotypes being compared and multiple tanks were used as replication. Plant were randomized within the tank and tanks were randomized within the chamber. For the quantitative PCR experiments all treatments and genotypes were included within the same plate and multiple plates were used as replications.
Blinding	Roots length were measured by a technician without knowledge of genotype of the plants (blind data collection). Data was then analyzed by the main author and the PI, who were not blinded to group allocation at the time of data analysis.

Reporting for specific materials, systems and methods

We require information from authors about some types of materials, experimental systems and methods used in many studies. Here, indicate whether each material, system or method listed is relevant to your study. If you are not sure if a list item applies to your research, read the appropriate section before selecting a response.

Materials & experimental systems

n/a	Involved in the study
☒	☐ Antibodies
☒	☐ Eukaryotic cell lines
☒	☐ Palaeontology and archaeology
☒	☐ Animals and other organisms
☒	☐ Clinical data
☒	☐ Dual use research of concern

Methods

n/a	Involved in the study
☒	☐ ChIP-seq
☒	☐ Flow cytometry
☒	☐ MRI-based neuroimaging
